# Anti-GQ1b Antibody Syndrome Presented as Locked-In Syndrome: A Rare Case Report

**DOI:** 10.7759/cureus.49866

**Published:** 2023-12-03

**Authors:** Kristen M D'Angelo, Jerilyn Williams, Laura Wu

**Affiliations:** 1 Neurology, University of Texas Medical Branch at Galveston, Galveston, USA

**Keywords:** locked-in syndrome, guillain-barre syndrome (gbs), anti-gq1b, miller fisher syndrome, bulbar palsy

## Abstract

Anti-GQ1b antibodies are considered a hallmark of Miller-Fisher syndrome (MFS), a rare variant of Guillain-Barré syndrome (GBS). The typical clinical presentation of MFS includes ophthalmoplegia, ataxia, and areflexia. Here, we present an unusual case of a 65-year-old man with acute-onset quadriplegia and bulbar weakness resembling locked-in syndrome. Imaging studies did not show structural lesions as a cause for his clinical symptoms. Nerve conduction studies showed severe axonal sensory-motor polyneuropathy. Serum studies were all negative except for a positive anti-GQ1b antibody. He was treated with plasmapheresis as MFS, with a quick improvement in muscle strength. Our case report provided further information on the clinical variation of anti-GQ1b syndrome. Physicians should pay more attention to unusual presentations of anti-GQ1b syndrome because, when it is recognized early with prompt treatment, patients are expected to have a good recovery.

## Introduction

Miller-Fisher syndrome (MFS), a rare variant of Guillain-Barré syndrome (GBS), is characterized by the acute onset of ophthalmoplegia, ataxia, and areflexia. The anti-GQ1b antibody was first discovered in typical Miller-Fisher syndrome in 1992 [1] and has been considered a hallmark of the diagnosis. Several other clinical syndromes, such as Bickerstaff brainstem encephalitis (BBE), acute ophthalmoplegia, and even GBS, have been reported to be associated with anti-GQ1b antibodies. Thus, anti-GQ1b antibody syndrome was proposed in later literature [2]. Anti-GQ1b syndrome is divided into six types according to the clinical manifestations: typical MFS, incomplete MFS, GBS, BBE, acute ophthalmoplegia, and pharynx-neck-brachial muscle weakness [3]. It is not uncommon that patients may have an incomplete clinical presentation for each syndrome or can present with overlapping syndromes.

Bulbar involvement is not part of the triad that MFS clinically presents with and is easily overlooked. When a patient presents with acute-onset severe weakness that includes bulbar involvement, it can resemble locked-in syndrome. Locked-in syndrome (LIS) is a condition often resulting from brain stem damage that commonly presents with quadriplegia and bulbar palsy with preserved vertical eye movements, blinking, and hearing. Patients with the incomplete form of LIS may have some preserved motor function, such as horizontal eye movement or facial expression. The most common cause of LIS is vascular complications to the brain stemming from hemorrhagic or ischemic strokes. Following that, traumatic injuries to the brainstem are the second most common cause of LIS [4]. Peripheral conditions may have clinical features similar to LIS, such as acute GBS, myasthenia gravis, and critical illness neuropathy/myopathy.

Here, we present a case of acute-onset quadriplegia with bulbar palsy resembling LIS secondary to a positive anti-GQ1b antibody and a good response to plasma exchange therapy.

## Case presentation

**Figure 1:**
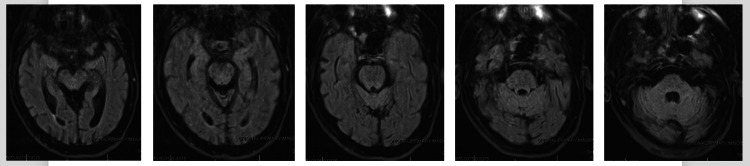
Brain MRI FLAIR The patient's brain MRI did not show abnormalities.

**Figure 2:**
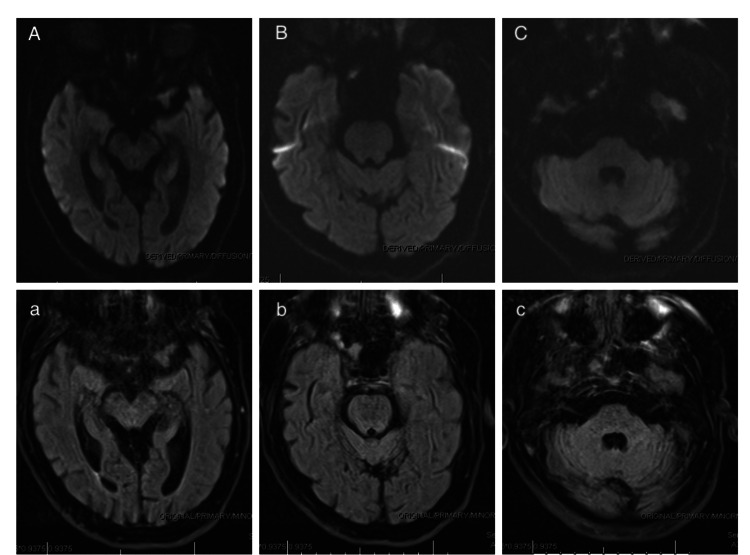
MRI DWI Upper A, B, C: DWI, lower a. b. c: FLAIR

**Figure 3:**
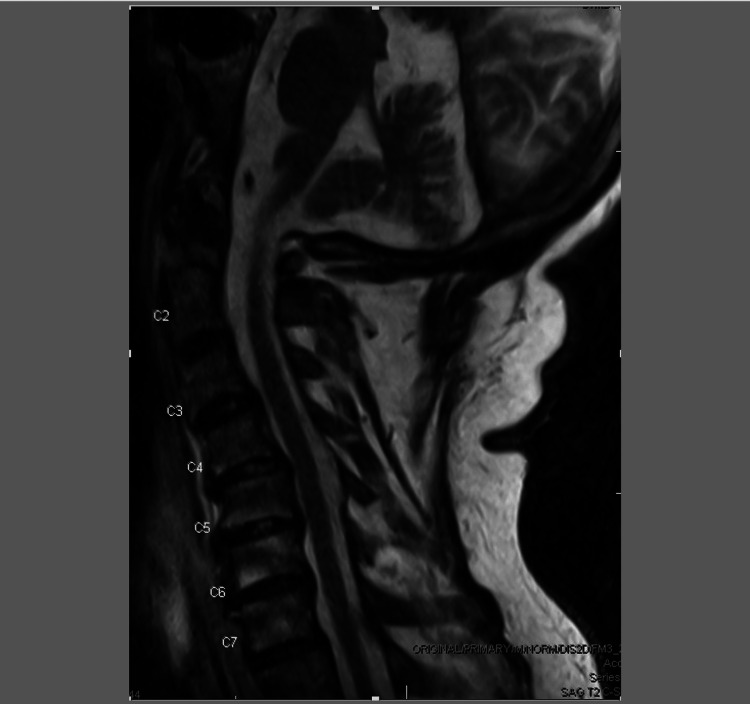
C-spine MRI T2 No abnormalities were noted on the patient's C-spine when imaged on an MRI.

**Figure 4:**
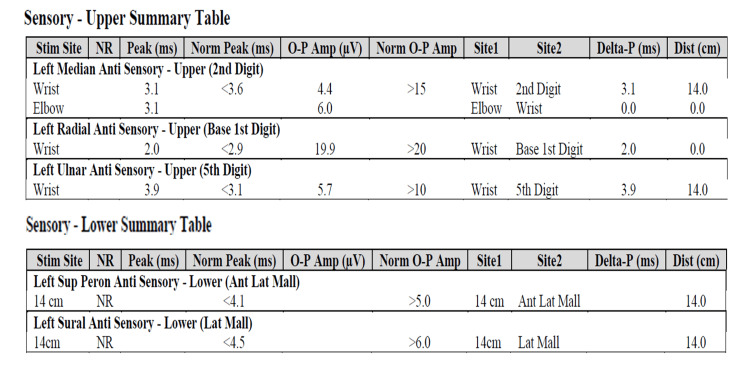
Upper and lower extremity sensory nerve summary table There is reduced amplitude shown in the sensory nerves of both upper and lower extremities, suggesting polyneuropathy with axonal loss.

**Figure 5:**
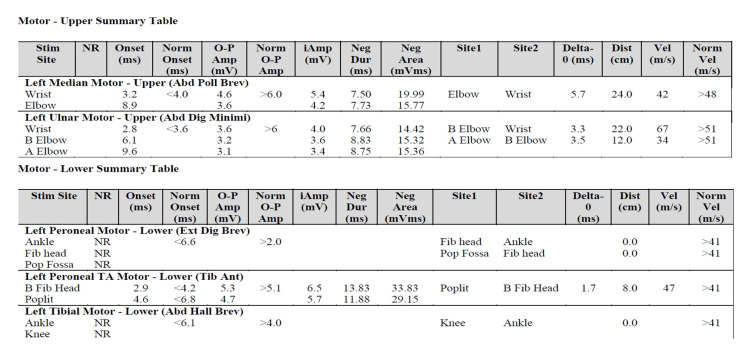
Upper and lower extremity motor nerve summary table Reduced amplitude in motor nerves in the upper and lower extremities.

**Figure 6:**
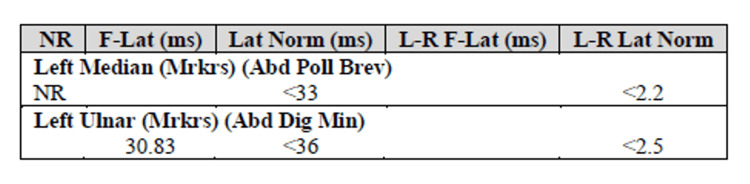
F-wave studies F-waves are absent. They are not recordable in the lower extremities and median nerve and are reduced in the ulnar nerve.

A 65-year-old man with a past medical history of HTN, alcohol abuse, liver cirrhosis, and pAfib was admitted to our hospital due to unresponsiveness secondary to *Escherichia coli* urosepsis and hepatic encephalopathy that required pressors and ventilatory support. His medical condition quickly improved within the first few days following intubation; however, he was still "unresponsive,” and neurology was consulted. On his first neurologic examination, he was able to move his eyes to sound stimulation and track providers with his eyes but was without verbal output. He was able to respond to some commands, including raising his eyebrows and blinking. His horizontal eye movements were intact while tracking the examiner. He was not able to show facial expressions and could barely open his mouth. All four limbs were flaccid. Patellar and Achilles reflexes were absent, but deep tendon reflexes on the upper extremities were preserved. The sensory exam was limited due to minimal communication. A cerebellar exam was unable to be obtained due to severe quadriplegia. Neuropathy labs were unremarkable (Table 1). Brain and cervical spine magnetic resonance imaging (MRI) was unremarkable, with no structural lesions such as pontine stroke or central pontine myelinolysis as possible causes for his quadriplegia (Figures 1-3). Nerve conduction studies demonstrated severe generalized sensory-motor polyneuropathy with axonal loss (Figures 4, 5). F-waves from lower limbs were not recordable due to absent sensory and motor M-responses (Figure 6). In the upper limbs, F-waves were absent in the median nerve and severely reduced persistence in the ulnar nerve. The antiganglioside antibody panel revealed a positive anti-GQ1b antibody with an anti-Gq1b titer of 60IV (ref: 0-50). All other antiganglioside antibodies, including anti-GM1, were negative. The patient was treated with five cycles of plasmapheresis. His muscle strength started to improve after the second plasma exchange, and all neurological symptoms continued to improve throughout the treatment. After completing the course, he was able to lift both legs against gravity. He was able to converse with simple verbal output. Upon discharge, he was able to sit up. His muscle strength continued to improve after discharge, and it was documented on a follow-up visit that he was able to stand up without assistance.

**Table 1:** Neuropathy work-up lab results A neuropathy lab workup is shown above. Several attempts at a lumbar puncture were unsuccessful.

Lab	Result	Units
B1	253	nmol/L
Folate	10	ng/mL
B12	922	pg/mL
TSH	0.09	mlU/L
Free T3	2.94	pg/mL
Free T4	1.20	ng/dL
Glucose	121	mg/dL
HCV Ab	Negative	--
HCV Semi-Quantitative	0.02	--
HBsAb	Negative	--
HBsAb Semi-Quantitative	0.00	mIU/mL
HBsAg	Negative	--
HBsAg Semi-Quantitative	0.09	--
HIV	Negative	--

## Discussion

The diagnosis of our case is challenging due to incomplete and overlapping manifestations of characteristic features of Miller-Fisher syndrome. Our patient did not present with ophthalmoplegia, and severe quadriplegia made it hard to examine or mask cerebellar dysfunction. Instead, the presentations of our patient resembled locked-in syndrome with preserved consciousness, intact eye range of motion, bulbar weakness, and quadriplegia. However, his imaging studies did not show any structural lesions in the brain, brain stem, or cervical spine to explain the clinical features. Areflexia of the lower extremities led us to suspect a peripheral etiology, which is supported by his nerve conduction study. Alongside the positive anti-GQ1b antibody, the diagnosis of Miller-Fisher syndrome was made, and plasma exchange started. The patient responded well to the treatment, and his muscle strength improved significantly in both limbs and the face.

Bulbar symptoms are not part of the triad of typical MFS but may be more common than we thought and are easily overlooked. In a case series report, seven out of 15 patients had bulbar palsy [5]. A wide range of clinical manifestations other than typical Miller-Fisher syndrome have been reported in patients with positive anti-GQ1b antibodies, including Bickerstaff brainstem encephalitis, Guillain-Barré syndrome, pharynx-neck-brachial muscle weakness, and acute ophthalmoparesis without ataxia [2]. A positive anti-GQ1b antibody can be identified in 86.5% of patients with MFS, 74% of patients with BBE, and 73% of patients with GBS with ophthalmoplegia, based on a previous report [6]. The case we presented here is quite unusual, with profound bulbar and limb weakness without ophthalmoplegia. Similar clinical presentations and nerve conduction studies can be seen in acute motor sensory axonal neuropathy (AMSAN), another variant of GBS. Positive antiganglioside antibodies can also be seen in AMSAN, but mostly anti-GM1b and anti-GD1a IgG, as well as anti-GM1 IgG. So far, no case report of AMSAN has presented with a positive anti-GQ1b antibody. Thus, our case supported more of the concept that the diagnosis of those overlap syndromes may be better based on antibodies rather than clinical manifestations.

Patients with anti-GQ1b antibody syndrome may have a proceeding infection, most commonly upper respiratory infection and acute gastroenteritis [7]. The neurological symptoms that occurred after an *Escherichia coli* infection, in our case, are uncommon. We only found one previous case report in the literature review with a similar *E. coli* infection [8]. Like most MFS patients, our patient responded well to treatment with a good recovery. The diagnosis of MFS or related disorders such as BBE can be challenging and still controversial due to the myriad of atypical and overlapping forms; thus, the term “anti-GQ1b antibody syndrome” has been introduced [2] to gain a better understanding of the etiological relationship among those patients. With the addition of our case report, it helps further expand the clinical spectrum of anti-GQ1b antibody syndrome.

## Conclusions

Diagnosis of MFS and associated disorders can be challenging due to multiple clinical variants of anti-GQ1b antibody syndrome. A positive serum antibody titer can aid in the diagnosis. Early recognition is important due to favorable responses to treatment.
